# A Rare Complication During Transcatheter Closure of Double Atrial Septal Defects With Incomplete Cor Triatriatum Dexter: A Case Report

**DOI:** 10.3389/fcvm.2021.815312

**Published:** 2022-02-24

**Authors:** Ping-Hong Chen, Yi-Ching Liu, Zen-Kong Dai, I-Chen Chen, Shih-Hsing Lo, Jiunn-Ren Wu, Yen-Hsien Wu, Jong-Hau Hsu

**Affiliations:** ^1^Department of Pediatrics, Kaohsiung Medical University Hospital, Kaohsiung, Taiwan; ^2^Department of Pediatrics, E-Da Hospital/I-Shou University, Kaohsiung, Taiwan; ^3^Department of Pediatrics, School of Medicine, College of Medicine, Kaohsiung Medical University, Kaohsiung, Taiwan

**Keywords:** atrial septal defect, cor triatiatum dexter, percutaneous catheterization, complication, heart ischemia

## Abstract

The cor triatiatum dexter is an embryologic remnant derived from the right atrium and totally separate from the right atrium. An incomplete cor triatiatum dexter (iCTD) means a partially obstructive remnant at the right atrium. It is usually formed by a remnant of the Eustachian valve (EV), Thebesian valve (ThV), or Chiari network (CN). This anatomic variant is usually asymptomatic but is often associated with other heart abnormalities including atrial septal defects (ASDs), and has the potential to hamper percutaneous heart procedures such as electrophysiological study or ASD closure. Herein, we report a rare complication, transient heart ischemia, in transcatheter closure of double ASDs in a 55-year-old woman with EV. This rare complication was thought to be caused by coronary sinus obstruction during device placement. The ischemic change was resolved spontaneously after we withdrew the device. For a second attempt, we adjusted the position of the device to avoid coronary sinus obstruction under transesophageal echocardiogram guidance and the device was smoothly deployed in a good position with a minimal residual shunt. This case suggests that anatomy details in percutaneous heart procedures are important, and this rare and dangerous complication, heart ischemia, should be identified immediately during the procedure.

## Introduction

Anatomic variants of the remnants of the right sinus venous valve are commonly observed in the right atrium (RA). They can be classified as normal structures or pathologic entities. Normal structures include the crista terminalis, taenia sagittalis, Chiari network (CN), Eustachian valve (EV), Thebesian valve (ThV), and coumadin ridge. An example of a pathologic entity is the cor triatriatum dexter (CTD) (1).

CTD is a rare congenital malformation of the RA in which the right venous valve is not resorbed, causing the RA to have two distinct chambers (2). The prevalence of CTD is rare, as it represents approximately only 0.025% of cases of all congenital heart diseases reported in previous literature (3). If the remnants that connect to the atrial septum are non-obstructive, it can be defined as incomplete CTD (iCTD) (4, 5). The symptoms of iCTD depend on the size of the septation between the two distinct chambers in the RA. When the septation is mild, most patients are often asymptomatic and these anomalies are mostly incidental findings during echocardiography. However, severe septation can cause generalized edema and even right-sided heart failure owing to inflow obstruction. It is often associated with other heart abnormalities, including atrial septal defect (ASD), tricuspid valve abnormality, and Ebstein anomaly.

Surgery is usually needed for ASD closure in patients with obstructive iCTD. In non-obstructive iCTD, few successful percutaneous procedures have been reported. These right atrial remnants may increase the difficulty to close the ASDs in percutaneous catheterization. More anatomic detail images, including transthoracic echocardiography (TTE), transesophageal echocardiography (TEE), computed tomography (CT), and magnetic resonance imaging (MRI), may be helpful in these patients before any percutaneous procedure (1).

## Case Presentation

**Figure 1:**
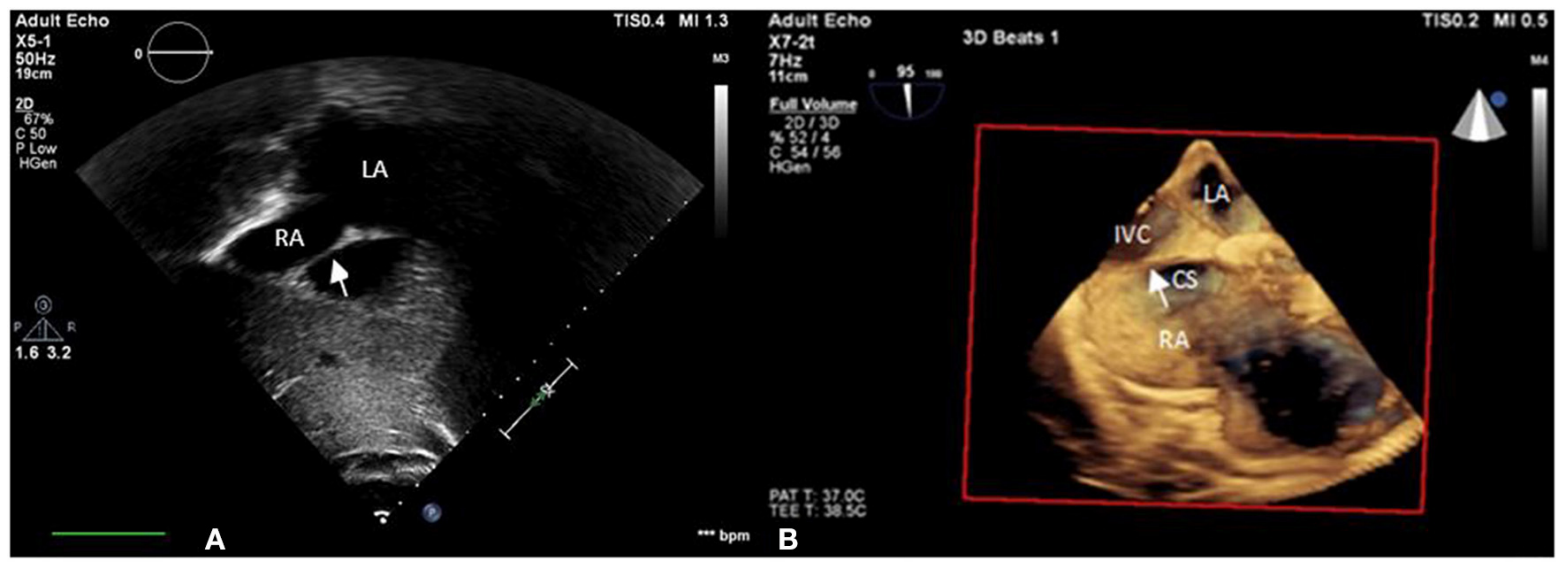
iCTD (arrow) at the connection of the right atrium and inferior vena cava is shown in **(A)** transthoracic echocardiogram (TTE) and **(B)** three-dimensional TEE. LA, left atrium; RA, right atrium; CS, coronary sinus; IVC, inferior vena cava.

**Figure 2:**
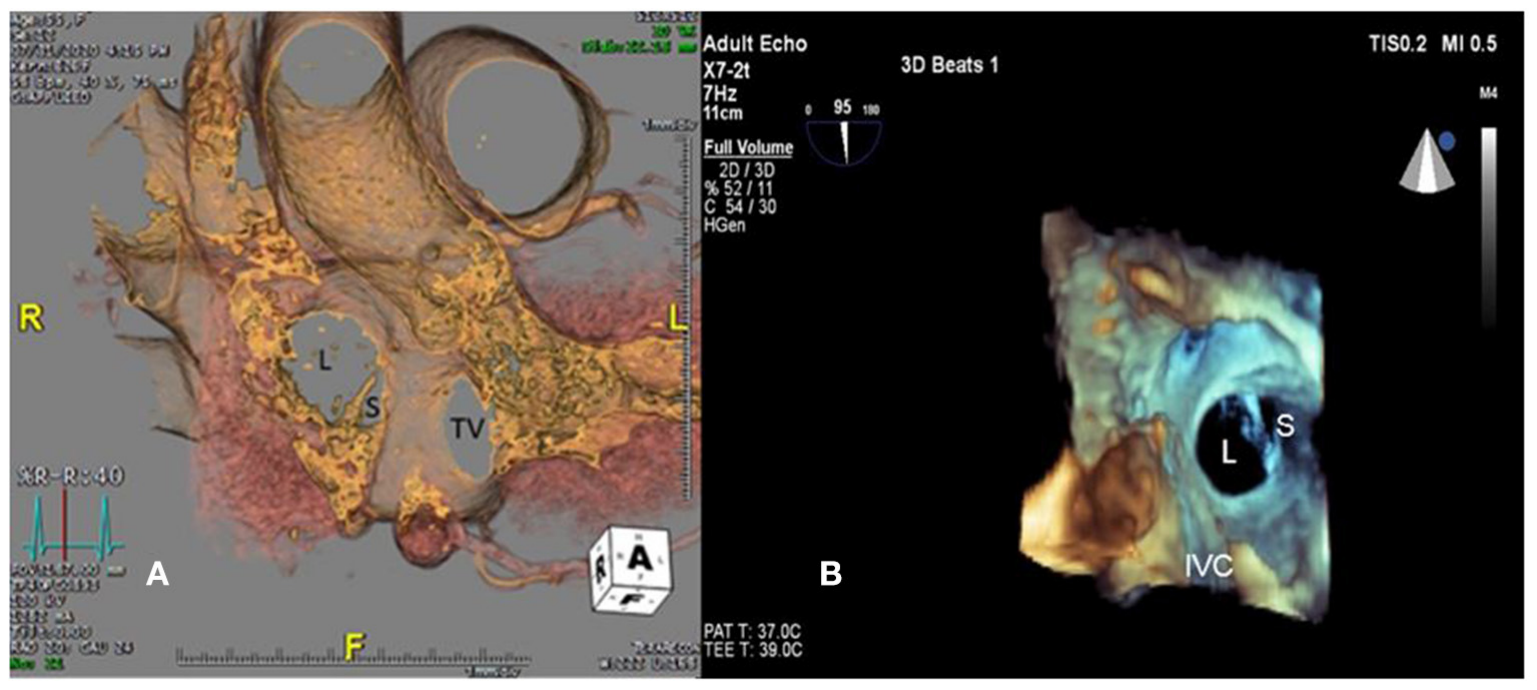
Large (L) and small (S) ASDs are revealed in **(A)** cardiac computed tomography and **(B)** three-dimensional transesophageal echocardiogram. TV, tricuspid valve.

**Figure 3:**
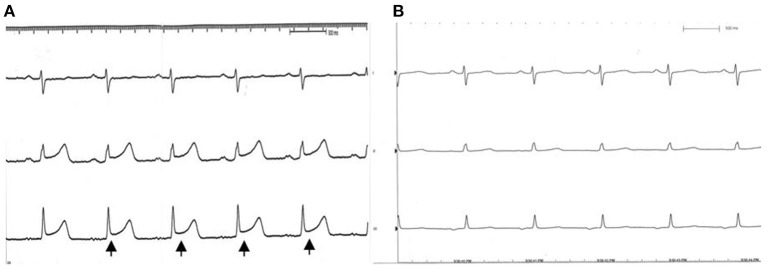
EKG during catheterization. **(A)** ST segment elevation (black arrow) is noted while the coronary sinus was obstructed by the occluder. **(B)** Recovery to sinus rhythm after we withdrew the device.

**Figure 4:**
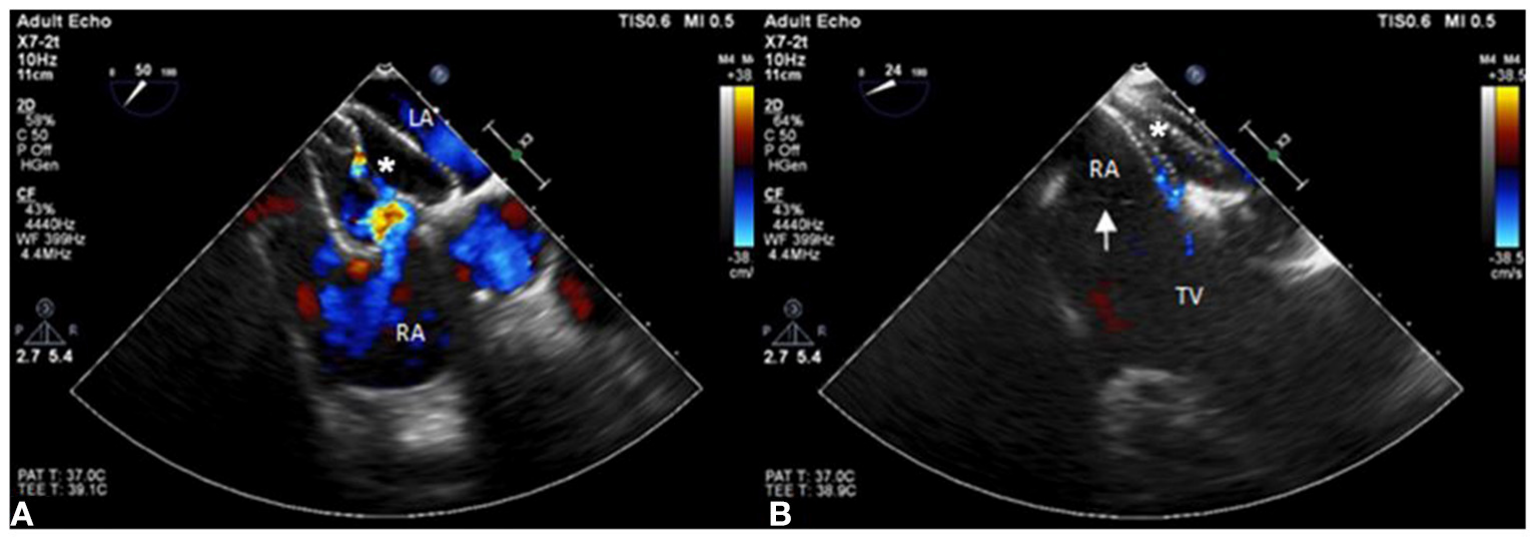
The position of the ASD occluder. **(A)** The position of the ASD device is initially incorrect with part of the incomplete cor triatiatum dexter clamped and many residual shunts noted under TEE. **(B)** The position of the ASD occluder is good with a minimal residual shunt and the incomplete cor triatiatum dexter (arrow) is free from clamping by the device.

A 55-year-old female patient came to our hospital with a complaint of chest discomfort for 2 days. The electrocardiogram (ECG) revealed an incomplete right bundle branch (iRBBB) without ST segment elevation. Troponin-I, creatine kinase (CK), and creatine kinase-MB (CK-MB) levels were in the normal range. Chest X-ray revealed right ventricle hypertrophy (RVH) with prominent pulmonary congestion. Two secundum type ASDs with membranous remnants at the right atrium were noted in TTE. TEE was arranged for the membranous remnants at the right atrium and revealed double ASDs with iCTD at the connection of the right atrium and inferior vena cava (Figures 1A,B). Cardiac CT showed double ASDs, which were 21.2 and 13.6 mm in size, with iCTD (Figures 2A,B). During catheterization, no pulmonary hypertension was found, and the size of the larger ASD under balloon sizing was 29.2 mm. The ratio of pulmonary flow to systemic flow was 1.9:1. Therefore, a 30-mm Lifetech device was chosen to close these two ASDs. We chose a 7 French sheath to pass the larger ASD from the RA to LA and deploy the device to close the ASDs without detachment. However, transient heart ischemia with presence of ST segment elevation (Figure 3A) was noted and coronary sinus obstruction by the device was thought to be the cause. The shape of the device was also not feasible and many residual shunts were noted. Partial involvement of iCTD in the process of device deployment, which resulted in the obstruction of coronary sinus, was determined (Figure 4A). Hence, we withdrew the device immediately and the ST segment elevation recovered (Figure 3B).

For the second attempt, a 7 French sheath passed the larger ASD using the same method as last time. The position of the device was adjusted more accurately than the first time to avoid iCTD involvement under TEE guidance. After deployment, the device was more stable than the first time, and no bradycardia or ST segment elevation were found on the electrocardiogram monitor. After device movement, we detached the device smoothly, and a minimal residual shunt without iCTD and tricuspid valve clamping was noted (Figure 4B). After the procedure, no complication was noted. Her chest tightness was improved and no residual shunt was noted in TTE during 6 months follow-up.

## Discussion

iCTD is an embryologic remnant deriving from the valve of the sinus venosus (3, 6). During embryogenesis, the right horn of the sinus venosus incorporates into the right atrium forming the smooth posterior portion. The left horn is the future coronary sinus (3, 7). This remnant is usually close to the ASD. As a result, it may influence the process of percutaneous closure of ASDs, especially the right disk of the device deployment (1, 2). The complications of ASD device malposition are residual atrial septal leak, bacterial endocarditis in the presence of a foreign body, and paradoxical embolism (8). Good preparation before the percutaneous catheterization is important to avoid these complications. Nowadays, TTE, TEE, cardiac CT, and MRI are valuable tools to help us find these remnants and recognize the morphology prior to the procedure. In the last 20 years, only a few case reports mentioned the effect of iCTD in percutaneous closure of ASDs (7, 9, 10). No clinical guideline describes the standard treatment of ASD closure with right atrial remnants. McMahon et al. reported four cases which had redundant EV in percutaneous closure of a secundum-type ASD (11). They used the steerable radiofrequency ablation catheter to deflect the EV toward the lateral wall of the RA. Three of these four patients underwent successful ASD closure by using this technique. Nevertheless, this skill is still not popular because few hospitals are equipped with steerable radiofrequency ablation.

Recently, McMahon CJ et al. reported the result of percutaneous ASD closure in six ASDs in children with iCTD. Two patients used the oversized device to close the ASD because of detachment. In two patients, it was difficult to trap the anterior border of the defect in the percutaneous closure. The other two patients were unable to receive percutaneous catheterization (5). This report reminds us of the difficulty of percutaneous catheterization with remnants in the RA. In our case, we chose a larger device to cover these two ASDs and open the right-side disc of the device more carefully under TEE guidance to avoid clamping the EV. During catheterization, the position of the RA remnants and the device are important to avoid device malposition and coronary sinus obstruction (12, 13). The unique point of our case is two ASDs with prominent iCTD, which is rarely reported. Besides, the occlusion of the coronary sinus causing the ischemic change during closure of the ASD has not been reported previously. Fortunately, we managed this case under TEE guidance by percutaneous catheterization and solved this rare but critical complication.

In conclusion, the importance of recognizing this remnant during anatomical study of ASDs is highlighted. This peculiar structure produces a diagnostic dilemma in echocardiography and it may bring about technical challenges and complications such as ischemic change during interventional procedures. Cardiac CT and MRI are important tools to differentiate between CTD, EV, ThV, or Chiari network. TEE guidance and device selection were important during intervention.

## Data Availability Statement

The original contributions presented in the study are included in the article/supplementary material, further inquiries can be directed to the corresponding author/s.

## Author Contributions

J-HH carried out the studies. Y-CL, Z-KD, and I-CC participated in collecting data. P-HC drafted the manuscript. J-RW and Y-HW helped to draft the manuscript. All authors contributed to the article and approved the submitted version.

## Conflict of Interest

The authors declare that the research was conducted in the absence of any commercial or financial relationships that could be construed as a potential conflict of interest.

## Publisher's Note

All claims expressed in this article are solely those of the authors and do not necessarily represent those of their affiliated organizations, or those of the publisher, the editors and the reviewers. Any product that may be evaluated in this article, or claim that may be made by its manufacturer, is not guaranteed or endorsed by the publisher.
